# Adjuvanted Influenza Vaccine Administered Intradermally Elicits Robust Long-Term Immune Responses that Confer Protection from Lethal Challenge

**DOI:** 10.1371/journal.pone.0010897

**Published:** 2010-05-28

**Authors:** Maria del P. Martin, Shaguna Seth, Dimitrios G. Koutsonanos, Joshy Jacob, Richard W. Compans, Ioanna Skountzou

**Affiliations:** 1 Department of Microbiology and Immunology and Emory Vaccine Center, Emory University School of Medicine, Atlanta, Georgia, United States of America; 2 MDRNA, Inc., Bothel, Washington, United States of America; University of Georgia, United States of America

## Abstract

**Background:**

The respiratory illnesses caused by influenza virus can be dramatically reduced by vaccination. The current trivalent inactivated influenza vaccine is effective in eliciting systemic virus-specific antibodies sufficient to control viral replication. However, influenza protection generated after parenteral immunization could be improved by the induction of mucosal immune responses.

**Methodology/Principal Findings:**

Transcutaneous immunization, a non-invasive vaccine delivery method, was used to investigate the quality, duration and effectiveness of the immune responses induced in the presence of inactivated influenza virus co-administered with retinoic acid or oleic acid. We observed an increased migration of dendritic cells to the draining lymph nodes after dermal vaccination. Here we demonstrate that this route of vaccine delivery in combination with certain immunomodulators can induce potent immune responses that result in long-term protective immunity. Additionally, mice vaccinated with inactivated virus in combination with retinoic acid show an enhanced sIgA antibody response, increased number of antibody secreting cells in the mucosal tissues, and protection from a higher influenza lethal dose.

**Conclusions/Significance:**

The present study demonstrates that transdermal administration of inactivated virus in combination with immunomodulators stimulates dendritic cell migration, results in long-lived systemic and mucosal responses that confer effective protective immunity.

## Introduction

Influenza infection and related complications result in thousands of hospitalizations and deaths worldwide every year. In the United States, there are currently two influenza vaccines licensed: a trivalent inactivated influenza vaccine (TIV) and the live attenuated influenza vaccine. The TIV induces mainly systemic strain-specific humoral responses while the intranasally administered live attenuated influenza vaccine generates mucosal humoral responses, but its use is limited to people between the ages of 2–49. A major hurdle in influenza prevention is its frequent antigenic change, which evades the host's acquired immunity[1], [2] and requires annual vaccination particularly of high-risk individuals. Therefore, alternative vaccine formulations, adjuvantation and routes of delivery are being investigated to create a more efficacious vaccine that would induce long-lived mucosal and systemic immune responses with broader cross-protection.

The skin is an immunologically active organ[3] where large numbers of antigen presenting cells (APCs), mainly Langerhans cells and dermal dendritic cells, reside. These populations form an integral part of the innate immune system, which upon antigen stimulation can prime and provide an amplified signal to the cells of the adaptive immune system[4]. The presence of APCs in high density and the skin accessibility make it an ideal target for vaccine delivery. APCs upon antigen uptake mature in response to inflammatory signals and migrate to the regional draining lymph nodes where they present antigen to T and B cells and initiate the adaptive immune responses[4], [5], [6], [7], [8].

Transcutaneous immunization (TCI) is a needle-free approach that involves the application of vaccine and often adjuvant to the skin surface. It is a simple, cost effective and fairly safe vaccine delivery method that may offer the additional advantages of self-administration. TCI successfully generates immunity not only with soluble proteins but also with large molecules such as particulate antigens despite the tight structure of the epidermis[9], [10], inducing mucosal and systemic immune responses as well as protection against viral infection[11], [12], [13], [14], [15], [16], [17], [18]. We have previously demonstrated that retinoic acid, oleic acid and cholera toxin as immunomodulators enhanced the magnitude of the immune response to transdermal [18]. Their capacity to increase the antigenic response when applied through the dermis demonstrate that they serve as bona fide adjuvants. A highly effective adjuvant should enhance both the magnitude and duration of the immune response against a particular pathogen[19].This principle is fundamental for protection against pathogens encountered long after immunization. In the present study we investigated these fundamental properties of transdermal vaccination and we report the effect of these immunomodulators on the duration of the immune response and their efficacy in generating protective immunity.

## Materials and Methods

### Reagents

Cholera toxin (CT) and oleic acid (OA) were purchased from Sigma-Aldrich (St. Louis, MO) and retinoic acid (RA) from Alexis Biochemicals (San Diego, CA). Purified mouse IgG, IgG1, and IgG2a antibodies were obtained from Southern Biotech (Birmingham, AL). ELISPOT reagents were purchased from BD-PharMingen and ELISA reagents from eBiosciences (San Diego, CA). Stable diaminobenzidine (DAB) was obtained from Research Genetics (Carlsbad, CA) and Tegaderm patches from 3M (Minneapolis, MN). Receptor-destroying enzyme was purchased from Roche Diagnostics (Indianapolis, IN). All H-2^d^–restricted Class I and II peptides were synthesized by the Emory University Peptide Facility. H-2^d^-restricted Hemagglutinin (HA) Class II peptides (SFERFEIFPKE, HNTNGVTAACSH, CPKYVRSAKLRM, KLKNSYVNKKGK, and NAYVSVVTSNYNRRF) and H-2^d^-restricted HA class I peptide (LYEKVKSQL) were used at 1 µg/m. Nucleoprotein (NP) H-2^d^-restricted class I peptide (TYQRTRALV, was used at 0.5 µg/ml and a pool of three H-2^d^-restricted class II peptides (FWRGENGRKTRSAYERMCNILKGK, RLIQNSLTIERMVLSAFDERNK, and AVKGVGTMVMELIRMIKRGINDRN) at 1 µg/ml[20], [21].

### Virus preparation

Influenza virus was prepared by inoculation of 10- or 11-day old embryonated hen's eggs with 2-5 HA units/egg of A/PR8/34 (PR8, H1N1). The hemagglutination (HA) activity was determined as previously described[22]. The virus was purified from allantoic fluid in discontinuous sucrose gradient as previously described [18]. Virus purification was followed by inactivation with formalin (0.01%) (vol/vol) for 72 hrs at 4°C, and dialyzed against PBS as described (ref). The protein concentration of the inactivated virus stock was determined by Bio-Rad Protein Assay. Inactivation was confirmed by inoculation of the virus into 10-day-old embryonated hen's eggs and plaque assay[23].

### Immunization

The dorsal caudal surface of twelve female BALB/C mice per group (Charles River Laboratory) was prepared for TCI as previously described [18]. RA and OA were co-administered with PR8 at concentrations of 50 µg/100 µl in 70% ethanol and 1% (w/v) in 70% ethanol respectively over an area of 1 cm^2^. Fifty µl of inactivated PR8 (100 µg) or with CT (100 µg in 50 µl) was applied to the skin and covered with a Tegaderm patch. Three TCI doses were administered 3 weeks apart. A group of mice were intranasally (i.n.) immunized at the same time intervals with inactivated PR8 (50 µg in 50 µl) as controls. Two separate cohorts were used for the evaluation of *in vitro* cellular immune responses 2 weeks after the 3d immunization dose immunization and for the duration of the humoral responses 12 weeks after the completion of the vaccination regimen. The animal protocols were performed in accordance with Emory's Institutional Animal Care and Use Committee guidelines.

### Influenza virus challenge

To determine post-challenge survival rates and perform immune assays, mice were challenged 12 weeks post-immunization by intranasal instillation of 5XLD_50_ or 20xLD_50_ of live mouse adapted A/PR/8/34 virus in 25 µl and monitored for morbidity based on body weight loss for 10 days. For lower infectious doses used in Table 1, the same mouse adapted A/PR/8/34 was used i.n. at 1xLD_50_ and 2xLD_50_ (300 and 600 PFU respectively). A mouse weight loss of over 25% was used as the experimental end-point. The survival data were reported using the Kaplan-Meier method. The mice that survived the infection were bled at the end of the monitoring period to assess the antibody response.

**Table 1:** Serum influenza-specific IgG titers.

Groups	IgG (ng/ml)
PBS	93.7±31.8
PR8	2043.0±131.1
PR8-OA-RA	3170.0±262.7^a^
PR8-CT	3884.3±111.1^a^
PR8-CT-RA	6205.9±432.9^a^^,^ b ^,^ c
PR8-CT-OA	6599.4±881.6^a^^,^ b
PR8 i.n.	7598.4±705.8^a^^,^ b ^,^ c
1xLD_50_	4785.9±384.3
2xLD_50_	8104.5±2087.1

Serum anti-influenza IgG antibody titers. Serum influenza specific IgG titers (ng/ml). The immunized mice (6 animals per group) were bled 2 weeks after immunization. Two groups of naïve mice were infected with 1xLD_50_ and 2xLD_50_ of live A/PR/6/34 and bled 8 days after infection. PBS, mock immunized mice with PBS were used as a negative control. TCI groups; PR8, inactivated influenza virus; PR8-OA-RA, virus co-administered with OA and RA; PR8-CT, inactivated influenza virus plus CT; PR8-CT-RA, inactivated influenza with RA and CT and; PR8-CT-OA, inactivated influenza virus and co-administration of OA and CT; PR8 i.n., intranasal immunization with 50 µg of inactivated virus as a positive control. Data shown are average and standard deviation (S.D.) from 6 mice per group. Statistics;

a: p<0.05 comparing all groups to the PR8 alone group,

b: <0.05 comparing adjuvanted groups to PR8-CT group (PR8-CT-RA, PR8-CT-OA, PR8-OA-RA) and

c: p<0.05, comparing all immunized groups to 1xLD_50_ infected group.

### Evaluation of humoral immune responses

Influenza-specific IgG, IgG1, IgG2a and IgA titers were determined in the sera and bronchoalveolar lavage fluid (BALF) of mice by enzyme linked immunosorbent assay (ELISA) as previously described[24].

### Neutralization assay

Sera were serially diluted and mixed with 100 pfu of PR8 virus, added to a MDCK cell monolayer and incubated for 1 hr at 37°C. The inoculum was removed, wells were overlayed with DMEM agar and incubated for 4 days at 37°C in a 5% CO2 humidified incubator. Then, plates were fixed with 0.25% gluteraldeyde and stained with 1% crystal violet in 20% ethanol, and plaques were counted[25], [26]. Neutralizing antibodies titers were determined as the reciprocal of the serum dilution that decreased by 50% the number of plaques formed by the live virus.

### Evaluation of cellular immune responses

Cellular immune responses were assayed by ELISPOT of spleen cells prepared from mice two weeks post immunization. 0.5–1.0×10^6^ splenocytes/well in complete RPMI 1640 medium (cRPMI: with 10% FBS, 10 mM L-glutamine, nonessential amino acids, HEPES buffer, and penicillin/streptomycin) were cultured for 36 hours in the presence of 0.5–1 µg/ml peptide stimulants, as described[27]. The number of cytokine producing lymphocytes was determined as described[24]. Briefly, multiscreen 96-well filtration plates were coated with IL-4 and IFN-γ capture antibodies at 4°C overnight and were blocked with 10% FBS in RPMI for 2 hr at 37°C. Splenocytes (1.0×10^6^/200 µl) were mixed with either HA immunogens (H-2^d^-restricted HA Class II peptides or one H-2^d^-restricted HA class I peptide) or NP immunogens (NP class II peptides or NP class I peptide) and plated in duplicates for 36–40 hrs. Biotinylated anti-mouse IL-4 or IFN-γ antibodies, streptavidin-HRP (horse-radish peroxidase) and DAB were used for detection. Spots were counted in an ELISPOT reader (Cellular Technology, Shaker Heights, OH).

### Antibody-secreting cell analysis

Virus-specific antibody-secreting cells (ASC) in the lungs and spleens of mice were evaluated at day 4 post-challenge (with 5xLD_50_ PR8) by a modification of the ELISPOT assay[28]. Briefly, 96-well plates were coated overnight at 4°C with 4 µg/ml of inactivated PR8 virus, washed and blocked for 2 h with 10% FBS. Single cell suspensions (1×10^6^/well) were plated and incubated at 37°C for 18 h. Positive spots were labeled with goat anti-mouse IgG HRP (Southern Biotech) and developed with DAB substrate. An ELISPOT reader was used to enumerate samples in triplicates and values were reported as the mean ASCs per 10^6^ cells.

### Cell trafficking

Inguinal lymph nodes were collected from sacrificed mice 2.5 days after application of the antigen ± adjuvant(s) to the dorsal skin. Single cell preparations were prepared as described [29] and stained with rat anti mouse CD205 Alexa 488 (Serotec), anti-CD11c PE, anti-CD11b APC and anti-CD8a+ PercP (BD-Pharmingen). Triple positive populations were considered dendritic cells and the percentage of total gated CD11c+/CD11b+/CD205+ cells was analyzed using FlowJo software.

### Statistics

We used one way or two way ANOVA test for comparison of parameters between groups and a value of p≤0.05 was considered significant. Unless otherwise stated, data are representative of at least three independent experiments.

## Results

### Humoral response enhancement by immunomodulators

**Figure 1:**
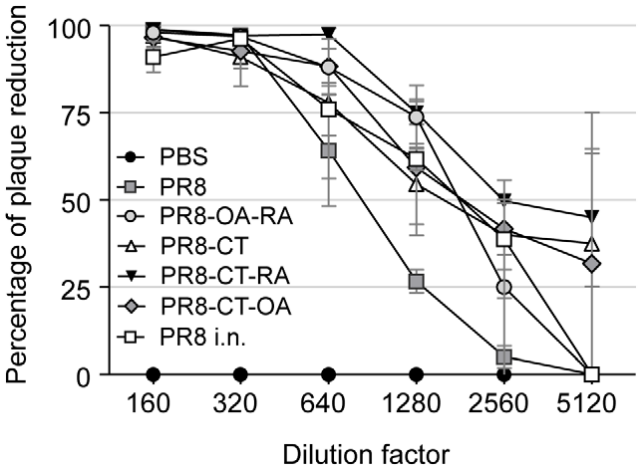
Neutralizing antibody titers after vaccination. Virus-neutralizing antibody activities in sera collected 2 weeks after immunization. Dilutions of sera were incubated with approximately 100 plaque forming units of PR8 virus for 1 hr at room temperature, applied to monolayers of confluent MDCK cells, and a standard plaque reduction assay was performed. Representative data are shown from at least three independent experiments. Data shown are average and standard error of mean (SEM) from 6 mice per group. Groups are as described in legend of Table 1.

We investigated whether mice transcutaneously immunized with influenza virus generated antibody titers similar to those observed after an influenza viral infection. Groups of mice received either inactivated PR8 virus alone (PR8) or combined with RA and OA (PR8-OA-RA), CT (PR8-CT), CT and RA (PR8-CT-RA) or CT and OA (PR8-CT-OA) and were compared to groups of mice that survived infection by intranasal instillation of 1xLD_50_ or 2xLD_50_ of mouse adapted PR8 virus. To assess the efficacy of TCI we used as control group immunized mice immunized intranasally with formalin inactivated influenza virus (PR8 i.n.)[30]. The mice that survived the challenge were tested 2-weeks post-infection for serum PR8-specific IgG antibodies and the titers were compared to those from immunized mice. The co-administration of RA or OA with PR8 and CT induced comparable antibody titers to the group infected with 2xLD_50_, indicating that TCI with inactivated influenza virus is capable of eliciting strong immune responses at similar levels to those obtained following virus infection (Table 1). In particular, the addition of retinoic acid to the PR8-CT regimen induced statistically significant higher antibody titers when compared to the group infected with 1xLD_50_ (P = 0.01). To confirm the generation of functional antibodies, we assayed the potential of the sera obtained from the immunized groups to neutralize viral replication *in vitro*. The highest neutralizing antibody titers were observed in the PR8-CT-RA group as seen by the 50% plaque reduction at a 2,560 serum dilution. The rest of the adjuvanted groups demonstrated 50% virus neutralization at a 1,280 dilution whereas the PR8 group showed the lowest neutralizing titers (640 dilution) (Figure 1). Some correlation was observed between the percent survival and the neutralizing titers. Although the PR8-CT and PR8-CT-OA groups were less effective than the PR8-CT-RA group in virus neutralizing activity (40% vs 50% plaque reduction at 2,560 dilution) their differences were not statistically significant. These data support the trend observed that when retinoic acid is used in combination with cholera toxin enhances the protective immune response.

**Figure 2:**
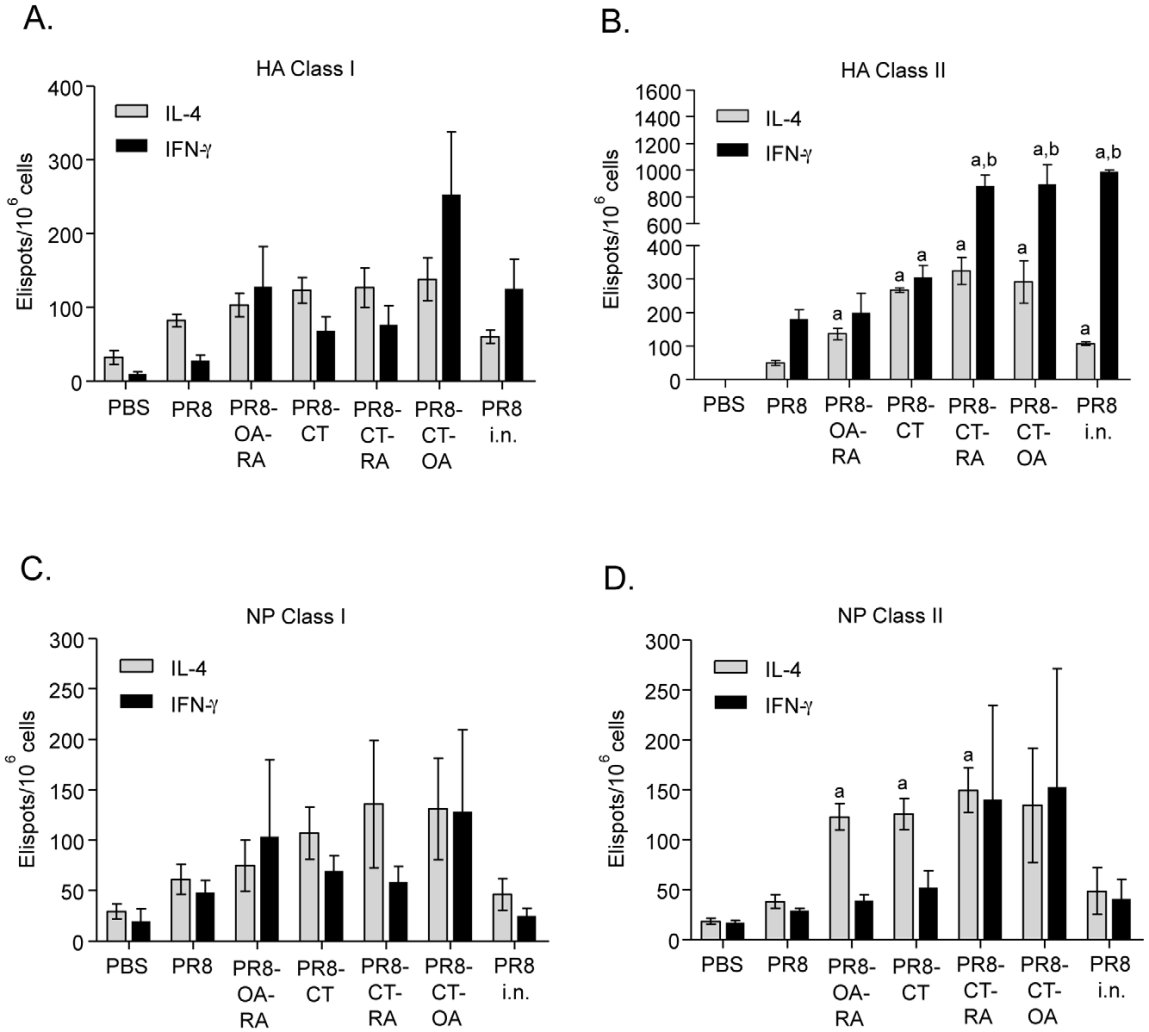
IL-4 and IFN-γ secreting cells in response to inactivated influenza antigen. The cellular immune responses were analyzed in splenocytes by ELISPOT for IL-4 and IFN-γ production 2 weeks after vaccination by re-stimulation with nucleoprotein (NP) and hemagglutinin (HA) class I (A,B) and class II (C,D) restricted peptides. Groups are as described in the legend of Table 1. Data are pooled from three independent experiments and are the average ± SEM for six mice per group. Statistical significance of the differences between groups in comparison was calculated by Student's test (p<0.05). Statistics; a: p<0.05 comparing all groups to the PR8 alone group, b: <0.05 comparing adjuvanted groups to PR8-CT group (PR8-CT-RA, PR8-CT-OA, PR8-OA-RA).

In order to assess the cellular immune responses generated after immunization we performed an *in vitro* re-stimulation of splenocytes from vaccinated mice with Class I and II H-2^d^ restricted hemagglutinin (HA) or nucleoprotein (NP) peptides. IFN-γ producing cells were increased by 3 to 8-fold in the PR8-OA-RA and PR8-CT-OA groups that were re-stimulated with Class I restricted HA peptides when compared to the TCI PR8 group. These differences were not statistically significant but the numbers of IFN-γ producing cells were similar to those generated in the i.n. immunized control group (Figure 2A). This observation suggests a higher generation of effector T cells by these adjuvants. The differences were more pronounced upon HA Class II restricted re-stimulation, after which the PR8-CT-RA and PR8-CT-OA groups showed a statistical significant increased in IFN-γ producing cells compared to both PR8 (P = 0.0004 and P = 0.0095, respectively) and PR8-CT groups (P = 0.0003 and P = 0.0197, respectively)(Figure 2B). The re-stimulation of splenocytes with HA Class I restricted peptides did not induce any statistically significant differences in the numbers of IL-4 producing cells among vaccinated groups, but showed enhanced responses in the PR8-CT-RA and PR8-CT-OA TCI groups when HA Class II restricted peptides were used (Figure 2A,B). Additionally, these groups showed a robust IFN-γ production similar to what observed after i.n. immunization. When we assessed the NP specific responses in vitro, due to the high variation in the number of IFN-γ producing cells we did not observed a distinction between the TCI groups. Similarly, the re-stimulation of splenocytes with NP Class I peptides lacked statistically significant differences in the numbers of IL-4 producing cells among the vaccinated groups (Figure 2C). In contrast, splenocytes re-stimulated with NP Class II peptides showed a marked increase of IL-4 producer cells in most adjuvanted groups as compared to the naïve or PR8 groups (P = 0.0013, 0.017 and 0.012 for PR-OA-RA, PR8-CT and PR8-CT-RA respectively) (Figure 2D).

Our data demonstrate that the PR8-CT co-administration of retinoic acid or oleic acid with induces marked increases in the numbers of IFN-γ producing CD4+ and CD8+ effector cells which correlate with effective viral clearance upon lethal challenge.

### Protective immunity to live virus challenge

**Figure 3:**
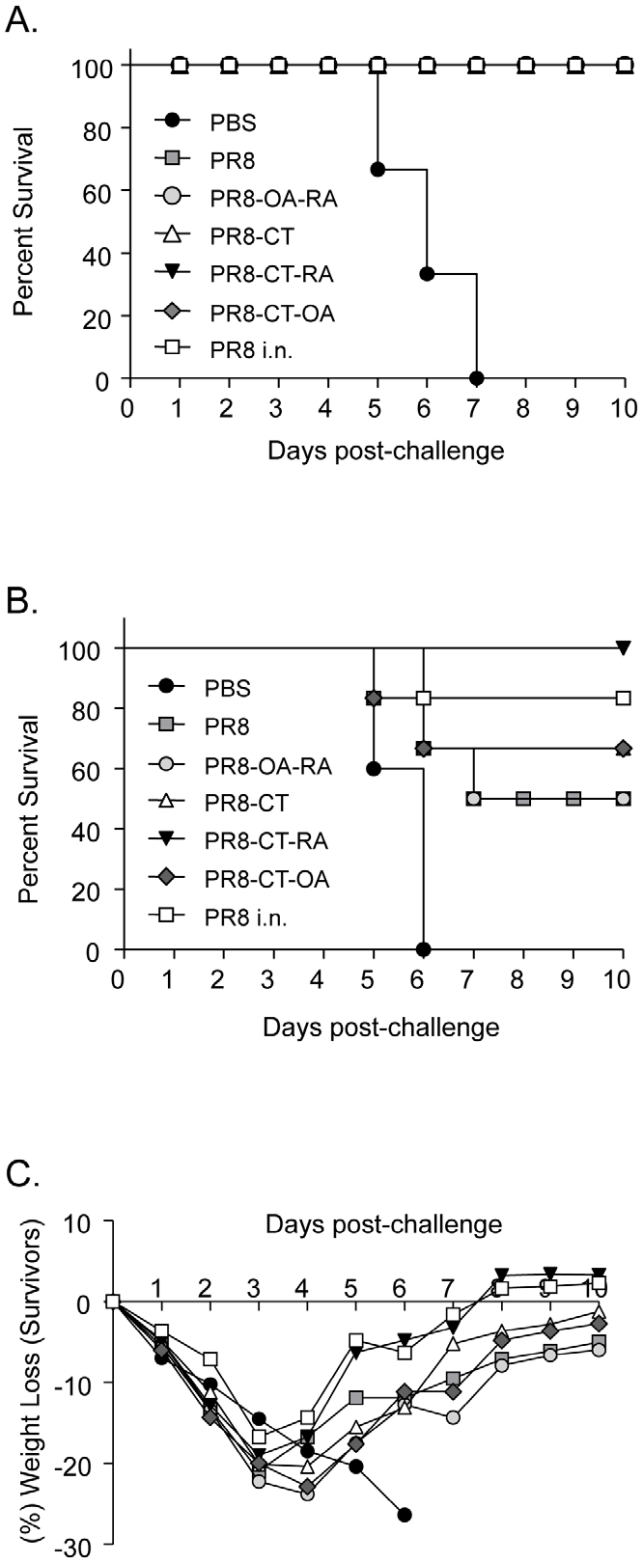
Protection of mice from lethal influenza virus challenge. Survival rates of immunized mice were monitored for 10 days after i.n. infection with mouse adapted PR8 virus. (A) Kaplan-Meier curve showing the percent of mice that survived a 5x LD_50_ PR8 virus challenge. (B) Percent of mice that survived a 20x LD_50_ PR8 virus challenge. (C) Percentage of body weight after i.n. challenge with 20x LD_50_ PR8 virus. PBS, PR8, PR8-OA-RA, PR8-CT, PR8-CT-OA, and PR8-CT-RA groups are as described in the legend of Table 1. Data represent the average of six mice per group for each challenge study.

Since TCI mice demonstrate robust humoral and cellular immune responses two weeks after vaccination, we evaluated the duration of the response by challenging the animals with mouse adapted influenza virus 12 weeks post-vaccination. The group of naïve mice infected with 5xLD_50_ of PR8 virus lost 25% of their body weight between days 5 and 7 and were euthanized. In contrast, TCI conferred protection with 100% survival in all PR8 vaccinated groups (Figure 3A). The TCI mice showed minimal weight loss and minimal to no morbidity signs during the evaluation period. To highlight the possible differences among the adjuvants used, we challenged a second cohort of mice with a 4-fold higher influenza lethal dose (20xLD_50_). We found that 100% of the mice immunized with PR8-CT-RA survived. Partial protection was observed in the remaining groups, whereas the naïve mice died between day 5 and 6 (Figure 3B). The percent of survival was tightly correlated with the percentage of body weight recovery suggestive of a milder disease in the groups with reduced mortality (Figure 3C).

### Systemic and mucosal humoral immune responses after lethal challenge

**Figure 4:**
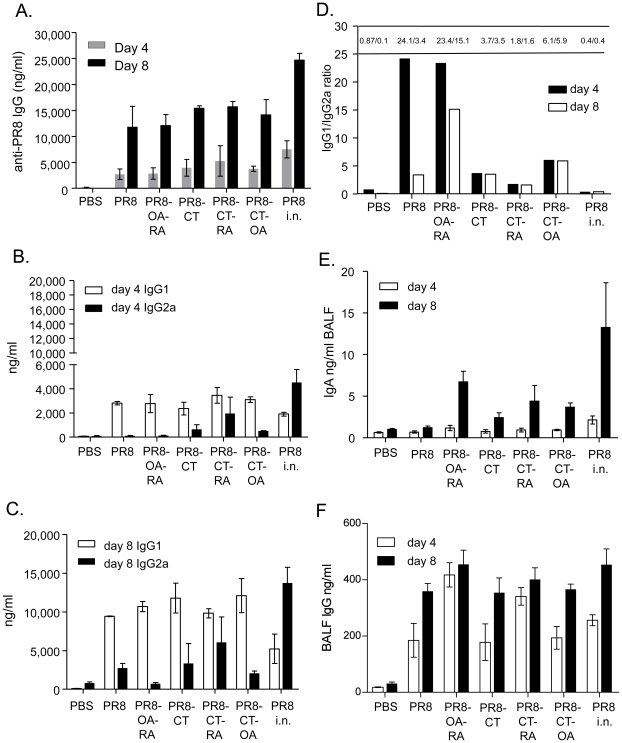
Antibody titers of post-challenged mice 4 and 8 days after intranasal infection with live A/PR/8/34 influenza virus. Total IgG anti-influenza titers determined by quantitative ELISA from immunized mice. Serum IgG1 and IgG2a isotype titers at day 4 (B,D) and day 8 (C,D) post-challenge. Ratios for IgG1/IgG2a were derived from the results in panels B and C. Mucosal IgA (E) and IgG (F) antibody titers by ELISA in bronchoalveolar lavage fluid (BALF) at day 4 and 8 after challenge. Data shown are average and standard error of mean (SEM) from 6 mice per group. Groups are as described in the legend of Table 1. Statistics; a: p<0.05 comparing all groups to the PR8 alone group.

Since transcutaneous immunization for most adjuvanted groups induced antibody titers as efficiently as live virus infection, we characterized the type of humoral response induced twelve weeks post-vaccination in mice challenged with 5xLD_50_ of mouse adapted PR8 virus. We studied the induction of serum PR8-specific IgG antibodies and the antibody isotype levels on days 4 and 8 after challenge. On day 4 post-challenge the differences observed in the IgG titers were not statistically significant, but we observed higher titers in the PR8-CT-RA relative to the other TCI groups (Figure 4A). By day 8, the IgG titers from all groups of TCI mice were increased similarly by 4-fold, reaching approximately 60% of levels observed by i.n. immunization (Figure 4A).

Differences in the systemic responses between the groups were observed in the antibody profiles generated. Immunoglobulin subtype IgG1 and IgG2a titers were evaluated due to their role in complement activation and because they are indicators of the cytokine environment in which the antibody response is generated [31], [32], [33]. We observed that at both time points after challenge the predominant responses against influenza antigen were of the IgG1 isotype (Figure 4B,C,D)[18] and that the use of CT, and particularly CT-RA, enhance the IgG2a isotype to the highest levels among the TCI groups. The presence of higher IgG2a titers was a common factor observed within the groups that showed a higher percent survival after lethal challenge. The generation of both IgG1 and IgG2a influenza specific antibodies in the groups that received CT and by intranasal immunization may have also contributed to the enhanced viral neutralization observed in these groups.

It has been reported that intranasal or dermal immunization induces effective mucosal immune responses, which have an important role in controlling viral replication in the respiratory system [34], [35], [36], [37]. In view of this, we assessed whether TCI enhanced the response in the bronchoalveolar lavage fluid (BALF) after challenge. The mucosal sIgA titers at 4 days post-challenge showed low levels of antibody production and with the exception of the PR8 i.n. group were similar to that observed in the PBS vaccinated control group (Figure 4E). Eight days post challenge we detected an increase in the sIgA levels in the TCI groups in which the immune enhancers OA and/or RA were co-administered. The increased IgA titers found by RA co-administration reached 50% of the titers induced by intranasal immunization (Figure 4E). Notably, in the RA treated groups the mucosal IgG titers increased substantially and appeared earlier (day 4) compared to the rest of the TCI groups suggestive of an RA-induced seroconversion in the mucosal tissue (Figure 4F).

**Figure 5:**
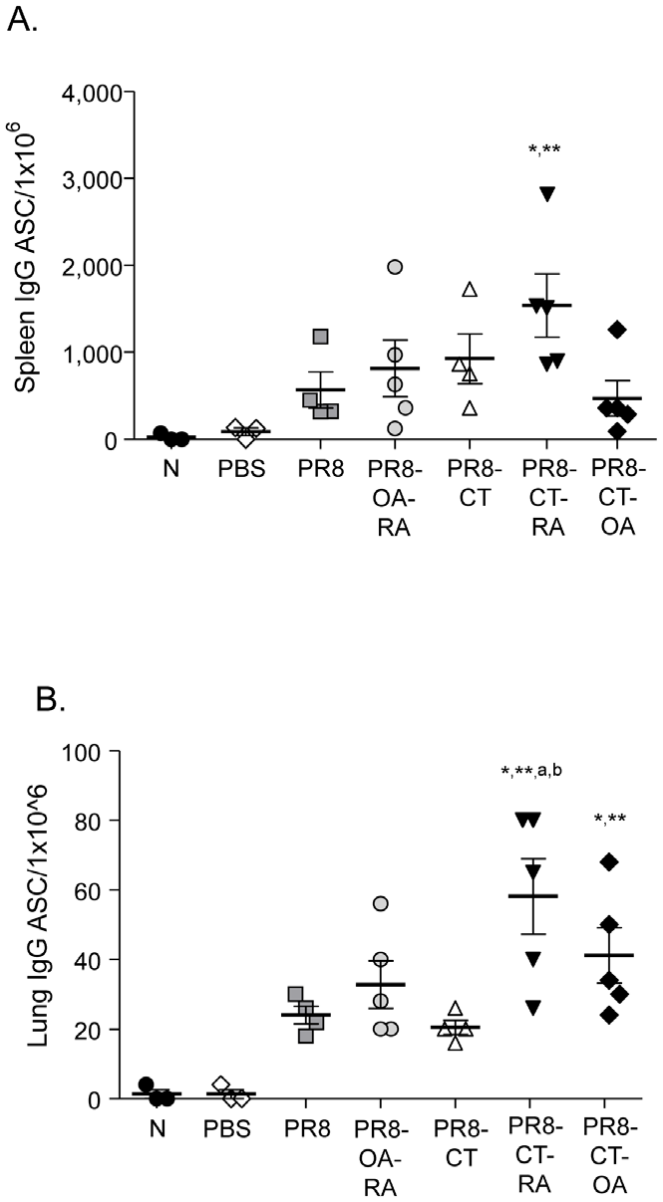
Influenza specific antibody secreting cells (ASC) in spleen and lungs of immunized mice. (A) Splenocytes and (B) single cell lung suspensions of vaccinated and control mice were assessed by ELISPOT 4 days post-challenge. Data shown are average and standard error of mean (SEM) from 5 mice per group. Groups are as described in the legend of Table 1. N: naïve (unimmunized) mice; PBS: mock immunized with PBS, challenged mice. Statistics; *: p<0.05 comparing all groups to the naive group;**: p<0.05 comparing all groups to the PBS alone group; a: p<0.05 comparing all groups to the PR8 alone group; b: <0.05 comparing adjuvanted groups to PR8-CT group (PR8-CT-RA, PR8-CT-OA, PR8-OA-RA).

To determine whether the co-administration of the adjuvants in TCI mice resulted in an increase of the number of antibody secreting cells (ASC) and their recruitment to the mucosal tissue, single cell suspensions from the spleen and lung tissue were used to identify influenza-specific ASC by ELISPOT assay. We found that TCI induces the generation of ASC in the spleen and that the presence of the adjuvants, in particular PR8-CT-RA, enhanced their total number (Figure 5A,B). In addition, influenza-specific ASC were found as early as 4 days post- infection in the lungs of the immunized mice. The number of lung ASC was significantly increased in the PR8-CT-RA group when compared to the mice vaccinated with PR8 or PR8-CT.

Taken together, these results demonstrate that the immune responses persisted 12 weeks post-TCI and that the levels of PR8-specific antibody titers correlate with an increase of total ASC in the lungs. Moreover this study shows that PR8 co-administration of RA or OA in combination with CT results in the enhancement of mucosal immune responses.

#### Enhanced mobilization of skin-associated dendritic cells (DC)

**Figure 6:**
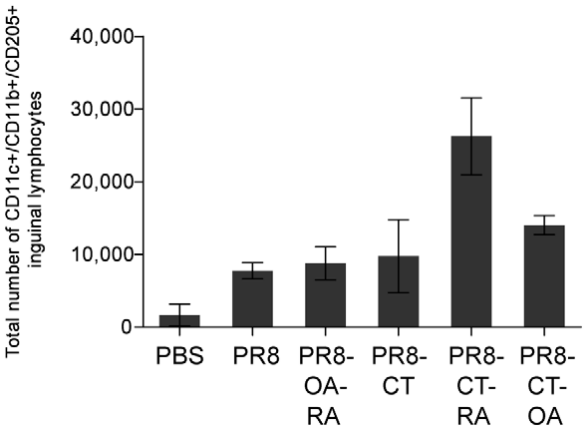
Dendritic cell mobilization to the lymph nodes upon TCI immunization. Dendritic cells were gated as triple positive populations (CD11c+/CD11b+/CD205+ cells). The total number of triple positive population/mouse lymph node was calculated and the data of each group of mice were averaged. Groups are as described in the legend of Table 1.

In order to better understand the mechanism of enhanced immune responsiveness observed, we determined whether TCI increased the migration of DC to the draining LN (Fig. 6). In view of the fact that DCs are equipped with multiple surveillance mechanisms and their activation is important to initiate the adaptive immune response, we evaluated migration of these cells to the draining LNs as an indication of increased antigen presentation and establishment of adaptive immunity [38], [39]. We administered inactivated influenza virus along with CT, OA or RA by TCI individually or in combination, and identified DC skin emigrants in the draining LNs by the expression of CD11c+/CD11b+/CD205+ surface markers [40], [41]. Mice immunized with PR8-CT-RA showed the highest numbers in LN of mice when compared with PR8. Interestingly, the PR8-CT-RA group increased the numbers of skin derived DC by 2.5-fold when compared to the PR8/CT indicating a synergistic effect (p<0.05). These data show that TCI promotes migration of skin-resident DC, and that the co-administration of CT and RA results in an enhanced stimulatory effect on this mobilization.

## Discussion

The present report demonstrates the potential of retinoic acid in combination with cholera toxin as potent immunomodulators in the protective responses induced by transcutaneous immunization with whole inactivated influenza virus. Although all TCI groups fully survived a 5xLD_50_ lethal dose, the only group that was completely protected against a high infectious dose was the PR8-CT-RA group. We observed that the use of cholera toxin in combination with retinoic acid or oleic acid as immunomodulators resulted in a seroconversion as robust as that observed following infection by live virus. The co-administration of CT and RA induced the highest neutralizing antibody titers, which is an important correlate of viral clearance and protection. Retinoic acid and OA also generated a robust cellular response as indicated by the increased numbers of IFN-γ secreting CD4+ and CD8+ T cells. The combination of CT with OA and mainly RA revealed that they were very effective as enhancers of mucosal and systemic humoral immune responses. The presence of RA in the PR8-CT group induced the highest systemic IgG2a titers among all groups and its addition accelerated the generation of mucosal IgG titers as seen in the PR8-CT-RA and PR8-OA-RA groups as early as 4 days post-challenge. The antibody titers induced by TCI correlated tightly with an increase of ASC. In particular, the PR8-CT-RA group showed the highest frequency of ASC in the mucosa which may have been critical in the 100% survival observed after challenge with a high lethal dose of mouse adapted virus.

Using the mouse model we found that TCI even in the absence of an adjuvant could induce robust recall B cell responses 12 weeks after immunization, which are considered critical in generating protective immunity[42]. The increase in serum IgG titers at day 4 post-infection suggests that this form of delivery generates persistent B cell responses that rapidly produce virus-specific antibodies. Although, the specific role of the antibody isotypes in influenza virus neutralization remains to be fully elucidated, we observed that in addition to the dominant IgG1 response normally described in dermal immunization[43], the co-administration of CT, RA and OA increased the production of IgG2a antibodies. Correspondingly, the groups that exhibited higher survival rates including the intranasally immunized group showed induction of influenza specific IgG2a antibodies.

The transdermal co-administration of CT and RA enhanced the mucosal antibody immune responses when compared to the rest TCI groups. In particular, influenza specific lung IgG titers were induced earlier and to comparable levels to the i.n. group. This finding is significant since protective mucosal responses are in general most effectively induced by mucosal immunization[44], [45], [46]. Importantly, the observed increase in antibody titers correlated with higher number of ASC in the lungs, which indicates an enhanced recruitment of B cells to the mucosal tissue. Based on these observations, we postulate that the enhanced lung humoral responses result from the RA effect in the imprinting of T and B cells to the mucosal tissue [47], [48], [49]. These results correlate with the complete protection of the PR8-CT-RA group against high infectious doses of influenza virus. Overall, we observed strong recall immune responses when CT, OA and RA were used as adjuvants or immunomodulators in transdermal vaccination.

The precise mechanism for inducing robust immune responses by an antigen delivered to the skin is not well understood. It has been postulated that the various dendritic cell populations residing in the epidermis, following immunization and antigen uptake, become activated and migrate to the regional lymph nodes where they present antigens to naïve T and B cells resulting in adaptive immune responses [11], [13], [16], [23], [50]. We observed that the co-administration of PR8-CT and OA or RA significantly increased mobilization of dendritic cells, which could influence T cell differentiation and cytokine secretions. We found high numbers of skin derived DCs in the draining LN implying their activation after antigen application [36]. This mobilization was particularly enhanced in the PR8-CT-RA group, corresponding to the group with a higher survival rate. Our data suggest that this stronger triggering of DC may have resulted in the effective adaptive immunity observed. Elucidating the downstream sequence of events and mediators involved in the observed enhancement of immune responses is of interest for further investigation.

In summary, the results support the conclusion that a transcutaneous influenza vaccine that employs a potent adjuvant as cholera toxin in combination with an immune enhancer such as RA induces increased humoral and cellular immune responses required for successful protection against influenza virus challenge. Most importantly, the present study suggests that the use of the appropriate adjuvant or immunomodulatory molecule could improve the efficacy of mucosal responses and could be utilized against other mucosal pathogens.

## References

[1] Webster RG, Bean WJ, Gorman OT, Chambers TM, Kawaoka Y (1992). Evolution and ecology of influenza A viruses.. Microbiol Rev.

[2] Bush RM, Bender CA, Subbarao K, Cox NJ, Fitch WM (1999). Predicting the evolution of human influenza A.. Science.

[3] Streilein JW (1983). Skin-associated lymphoid tissues (SALT): origins and functions.. J Invest Dermatol.

[4] Banchereau J, Steinman RM (1998). Dendritic cells and the control of immunity.. Nature.

[5] Romani N, Stingl G, Tschachler E, Witmer MD, Steinman RM (1985). The Thy-1-bearing cell of murine epidermis. A distinctive leukocyte perhaps related to natural killer cells.. J Exp Med.

[6] Sallusto F, Lanzavecchia A (1999). Mobilizing dendritic cells for tolerance, priming, and chronic inflammation.. J Exp Med.

[7] van Stipdonk MJ, Lemmens EE, Schoenberger SP (2001). Naive CTLs require a single brief period of antigenic stimulation for clonal expansion and differentiation.. Nat Immunol.

[8] Kaech SM, Ahmed R (2001). Memory CD8+ T cell differentiation: initial antigen encounter triggers a developmental program in naive cells.. Nat Immunol.

[9] Stingl G, Steiner G (1989). Immunological host defense of the skin.. Curr Probl Dermatol.

[10] Bos JD, Meinardi MM (2000). The 500 Dalton rule for the skin penetration of chemical compounds and drugs.. Exp Dermatol.

[11] Glenn GM, Scharton-Kersten T, Vassell R, Matyas GR, Alving CR (1999). Transcutaneous immunization with bacterial ADP-ribosylating exotoxins as antigens and adjuvants.. Infect Immun.

[12] Scharton-Kersten T, Yu J, Vassell R, O'Hagan D, Alving CR (2000). Transcutaneous immunization with bacterial ADP-ribosylating exotoxins, subunits, and unrelated adjuvants.. Infect Immun.

[13] El-Ghorr AA, Williams RM, Heap C, Norval M (2000). Transcutaneous immunisation with herpes simplex virus stimulates immunity in mice.. FEMS Immunol Med Microbiol.

[14] Chen D, Erickson CA, Endres RL, Periwal SB, Chu Q (2001). Adjuvantation of epidermal powder immunization.. Vaccine.

[15] Guerena-Burgueno F, Hall ER, Taylor DN, Cassels FJ, Scott DA (2002). Safety and immunogenicity of a prototype enterotoxigenic Escherichia coli vaccine administered transcutaneously.. Infect Immun.

[16] Guebre-Xabier M, Hammond SA, Epperson DE, Yu J, Ellingsworth L (2003). Immunostimulant patch containing heat-labile enterotoxin from Escherichia coli enhances immune responses to injected influenza virus vaccine through activation of skin dendritic cells.. J Virol.

[17] Berry LJ, Hickey DK, Skelding KA, Bao S, Rendina AM (2004). Transcutaneous immunization with combined cholera toxin and CpG adjuvant protects against Chlamydia muridarum genital tract infection.. Infect Immun.

[18] Skountzou I, Quan FS, Jacob J, Compans RW, Kang SM (2006). Transcutaneous immunization with inactivated influenza virus induces protective immune responses.. Vaccine.

[19] Pulendran B, Miller J, Querec TD, Akondy R, Moseley N (2008). Case of yellow fever vaccine–associated viscerotropic disease with prolonged viremia, robust adaptive immune responses, and polymorphisms in CCR5 and RANTES genes.. J Infect Dis.

[20] Gerhard W, Haberman AM, Scherle PA, Taylor AH, Palladino G (1991). Identification of eight determinants in the hemagglutinin molecule of influenza virus A/PR/8/34 (H1N1) which are recognized by class II-restricted T cells from BALB/c mice.. J Virol.

[21] Deng Y, Yewdell JW, Eisenlohr LC, Bennink JR (1997). MHC affinity, peptide liberation, T cell repertoire, and immunodominance all contribute to the paucity of MHC class I-restricted peptides recognized by antiviral CTL.. J Immunol.

[22] Compans RW (1974). Hemagglutination-inhibition: rapid assay for neuraminic acid-containing viruses.. J Virol.

[23] Enioutina EY, Visic D, Daynes RA (2000). The induction of systemic and mucosal immune responses to antigen-adjuvant compositions administered into the skin: alterations in the migratory properties of dendritic cells appears to be important for stimulating mucosal immunity.. Vaccine.

[24] Kang SM, Guo L, Yao Q, Skountzou I, Compans RW (2004). Intranasal immunization with inactivated influenza virus enhances immune responses to coadministered simian-human immunodeficiency virus-like particle antigens.. J Virol.

[25] Novak M, Moldoveanu Z, Schafer DP, Mestecky J, Compans RW (1993). Murine model for evaluation of protective immunity to influenza virus.. Vaccine.

[26] Sha Z, Compans RW (2000). Induction of CD4(+) T-cell-independent immunoglobulin responses by inactivated influenza virus.. J Virol.

[27] Oran AE, Robinson HL (2003). DNA vaccines, combining form of antigen and method of delivery to raise a spectrum of IFN-gamma and IL-4-producing CD4+ and CD8+ T cells.. J Immunol.

[28] Koutsonanos DG, del Pilar Martin M, Zarnitsyn VG, Sullivan SP, Compans RW (2009). Transdermal influenza immunization with vaccine-coated microneedle arrays.. PLoS ONE.

[29] Garg S, Oran A, Wajchman J, Sasaki S, Maris CH (2003). Genetic tagging shows increased frequency and longevity of antigen-presenting, skin-derived dendritic cells in vivo.. Nat Immunol.

[30] Sha Z, Kang SM, Compans RW (2005). Mucosal immunization of CD4(+) T cell-deficient mice with an inactivated virus induces IgG and IgA responses in serum and mucosal secretions.. Virology.

[31] Heyman B (2000). Regulation of antibody responses via antibodies, complement, and Fc receptors.. Annu Rev Immunol.

[32] Ronnelid J, Ahlin E, Nilsson B, Nilsson-Ekdahl K, Mathsson L (2008). Immune complex-mediated cytokine production is regulated by classical complement activation both in vivo and in vitro.. Adv Exp Med Biol.

[33] Moulds JM (2009). Introduction to antibodies and complement.. Transfus Apher Sci.

[34] Asahi Y, Yoshikawa T, Watanabe I, Iwasaki T, Hasegawa H (2002). Protection against influenza virus infection in polymeric Ig receptor knockout mice immunized intranasally with adjuvant-combined vaccines.. J Immunol.

[35] Gockel CM, Bao S, Beagley KW (2000). Transcutaneous immunization induces mucosal and systemic immunity: a potent method for targeting immunity to the female reproductive tract.. Mol Immunol.

[36] Belyakov IM, Hammond SA, Ahlers JD, Glenn GM, Berzofsky JA (2004). Transcutaneous immunization induces mucosal CTLs and protective immunity by migration of primed skin dendritic cells.. J Clin Invest.

[37] Holmgren J, Czerkinsky C (2005). Mucosal immunity and vaccines.. Nat Med.

[38] Ueno H, Schmitt N, Klechevsky E, Pedroza-Gonzalez A, Matsui T (2010). Harnessing human dendritic cell subsets for medicine.. Immunol Rev.

[39] Henri S, Guilliams M, Poulin LF, Tamoutounour S, Ardouin L (2010). Disentangling the complexity of the skin dendritic cell network.. Immunol Cell Biol.

[40] Henri S, Vremec D, Kamath A, Waithman J, Williams S (2001). The dendritic cell populations of mouse lymph nodes.. J Immunol.

[41] Shortman K, Liu YJ (2002). Mouse and human dendritic cell subtypes.. Nat Rev Immunol.

[42] Plotkin SA (2008). Vaccines: correlates of vaccine-induced immunity.. Clin Infect Dis.

[43] Anjuere F, George-Chandy A, Audant F, Rousseau D, Holmgren J (2003). Transcutaneous immunization with cholera toxin B subunit adjuvant suppresses IgE antibody responses via selective induction of Th1 immune responses.. J Immunol.

[44] Neutra MR, Kozlowski PA (2006). Mucosal vaccines: the promise and the challenge.. Nat Rev Immunol.

[45] Amorij JP, Meulenaar J, Hinrichs WL, Stegmann T, Huckriede A (2007). Rational design of an influenza subunit vaccine powder with sugar glass technology: preventing conformational changes of haemagglutinin during freezing and freeze-drying.. Vaccine.

[46] Li Z, Zhang M, Zhou C, Zhao X, Iijima N (2008). Novel vaccination protocol with two live mucosal vectors elicits strong cell-mediated immunity in the vagina and protects against vaginal virus challenge.. J Immunol.

[47] Manicassamy S, Pulendran B (2009). Retinoic acid-dependent regulation of immune responses by dendritic cells and macrophages.. Semin Immunol.

[48] Mora JR, von Andrian UH (2009). Role of retinoic acid in the imprinting of gut-homing IgA-secreting cells.. Semin Immunol.

[49] Eksteen B, Mora JR, Haughton EL, Henderson NC, Lee-Turner L (2009). Gut homing receptors on CD8 T cells are retinoic acid dependent and not maintained by liver dendritic or stellate cells.. Gastroenterology.

[50] Enioutina EY, Visic VD, Daynes RA (2000). Enhancement of common mucosal immunity in aged mice following their supplementation with various antioxidants.. Vaccine.

